# Supplementary material for: Strategically Altered Fluorinated Polymer at Nanoscale for Enhancing Proton Conduction and Power Generation from Salinity Gradient
Source: Membranes (Basel). 2022 Apr 1;12(4):395. doi: 10.3390/membranes12040395 (PMC9025533; doi:10.3390/membranes12040395)
Supplement: Supplementary file 1 [file membranes-12-00395-s001.zip › membranes-1634165-supplementary.pdf]

Supplementary information

Strategically altered fluorinated polymer at nanoscale for enhancing proton conduction and power generation from salinity gradient

Prem P Sharma[a, †], Rahul Singh[b, †], Syed Abdullah Shah[b], Cheol Hun Yoo[a], Albert S. Lee[c], Daejoong Kim[b,]* Jeong-Geol Na [a, *], Jong Suk Lee[a, *]

[a]*Department of Chemical and Biomolecular Engineering, Sogang University, 35, Baekbeom-ro, Mapo-gu, Seoul 04107, Republic of Korea.*
[b]*Department of Mechanical Engineering, Sogang University, 35, Baekbeom-ro, Mapo-gu, Seoul 04107, Republic of Korea.*
[c]*Korea Institute of Science and Technology, Hwarangno 14 gil-5, Seongbuk-gu, Seoul 02792, Republic of Korea*

*Corresponding author:
jongslee@sogang.ac.kr (J. S. Lee),
daejoong@sogang.ac.kr (D. Kim),
narosu@sogang.ac.kr (J-G Na)

† Equal contribution:

Number of pages: 19

Number of figures: 09

Number of tables: 04

Number of notes: 03

Supplementary Content:

1. Characterizations
2. Supplementary Note S1: Nanostructure estimation
3. Supplementary Note S2: Electrochemical and physicochemical characterization of the membrane
4. Supplementary Note S3: RED stack Assembly
5. Figure S1: small-angle X-ray scattering Guinier Plot
6. Figure S2: small-angle X-ray scattering log-log plot
7. Table S1: Diameter of nanochannel width and its corresponding q value for dry samples.
8. Table S2: Numerical value obtained from regions 1 and 2 of the log-log plot and Guinier analysis.
9. Figure S3: X-ray diffraction of the bulk nanoparticle and exfoliated nanosheet of graphitic carbon nitride.
10. Figure S4: SEM images of bulk and exfoliated g-C_3N_4, and TEM images of exfoliated g-C_3N_4.
11. Figure S5: EDS analysis of a particular region of the particle, and EDS analysis containing elemental composition for g-C_3N_4.
12. Figure S6: FT-IR spectra of pristine graphitic carbon nitride and sulfonated graphitic carbon nitride.
13. Figure S7: Fluorine-19 nuclear magnetic resonance spectroscopy (^{19}F- NMR)
14. Figure S8: Proton Nuclear magnetic resonance spectroscopy (^{1}H-NMR)
15. Figure S9: Water uptake vs Ion exchange capacity
16. Table S3: Summary of prepared cation-exchange membrane and commercially available perfluorosulfonic acid membrane materials properties.
17. Table S4. Comparison of commercially available membranes for reverse electrodialysis

1. Characterizations

Fourier-Transform Infrared Spectroscopy (FT-IR) spectra were measured using a Perkin-Elmer FT-IR Frontier instrument in the wavenumber range of 4000–400 cm^{-1}. The sulfonated polymer was characterized by ^{1}H NMR at 500MHz (Nuclear Magnetic Resonance Spectrometer) by choosing DMSO as solvent. ^{19}F NMR of the defluorinated polymer was recorded by Unity Inova 500 (Varian) NMR spectrometer. Surface morphology and size of nanosheets and membrane were evaluated by FE-SEM (JSM-7100F). Phase identification for both particle and semi-crystalline polymer backbone was determined by X-ray diffraction (Rigaku) with CuKα radiation (λ=1.54 Å). Stepwise weight loss of composite membranes and quantification of the actual amount of material was characterized by TGA analysis (Mettler-Toledo). EDS analysis and images with high resolution were recorded by TEM instrument, JEM-2100F, JEM-4010 (JEOL).

Supplementary Note 1

Nanostructure estimation

It is noted that if the distance between sample and CCD is small i.e. 1m, the 2θ angle's value is higher. Whereas, if the distance between sample and CCD is large, i.e. 4m, the 2θ angle value is low. Typically, two maxima observed from the SAXS plot, one at the down q side, represent the crystalline domain. The second one at a relatively higher q side represents the ionic nanochannel of polymer [1]. Hence, 4m length between CCD and the membrane is used to analyze the adequate size of inter-crystalline distance, and 1m length between CCD and the membrane is used to analyze the width of the nanochannel. The SAXS plot of pure PVDF-HFP and sulfonated-PVDF-HFP measured at 1m and 4m is used for estimating the ionic phase and inter-crystalline domain, respectively using Bragg distance equation: $d = 2\pi/q$; Where q is ($q = 4\pi/\sin\theta\ \lambda$) . It is noted that d-spacing is inversely proportional to the q value.

(a) SAXS measurement at 1m (distance between CCD and sample): Plots were used to evaluate the ionic nanochannel below ~10 nm.

(b) SAXS measurement at 4m (distance between CCD and sample): Plots were used to evaluate the inter-crystalline distance above ~10 nm.

Table S1 Diameter of nanochannel width and its corresponding q value for dry samples.

**Sample**	**q (A^{-1})**	**d* (nm)**
**Sulfonated-PVDF-co-HFP**	0.4	1.6
**Nafion**	0.15	4.1

Table S2. Numerical values obtained from regions 1 and 2 of the log-log plot and Guinier analysis.

**Sample**	**Double log plot**				**Guinier Analysis**		
	**region 1**	**R^2**	**region 2**	**R^2**	**Rg (A^{-1})**	**I(0)**	**qRg**
Membrane Temperature (25 ^oC)							
Pristine (PVDF-co-HFP)	-3	0.99	-2.95	0.93	>100	3.6	1.07
Sulfonated-PVDF-co-HFP	-3.2	0.97	-3.6	0.84	40	2.30	1.28
Nafion	-0.30	0.97	-1.36	0.99	9.16	3.22	0.14

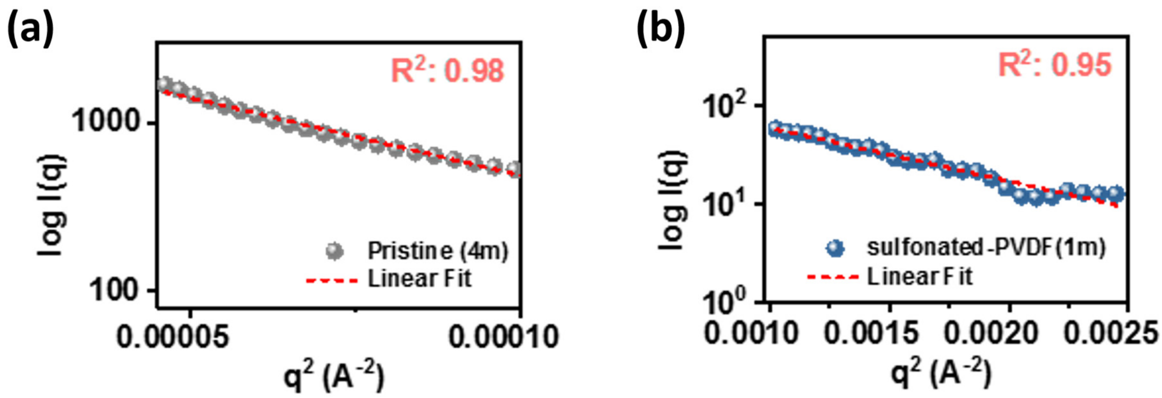

Figure S1. Guinier Plot (a) pristine PVDF-co-HFP membrane, (b) sulfonated-PVDF-co-HFP membrane.

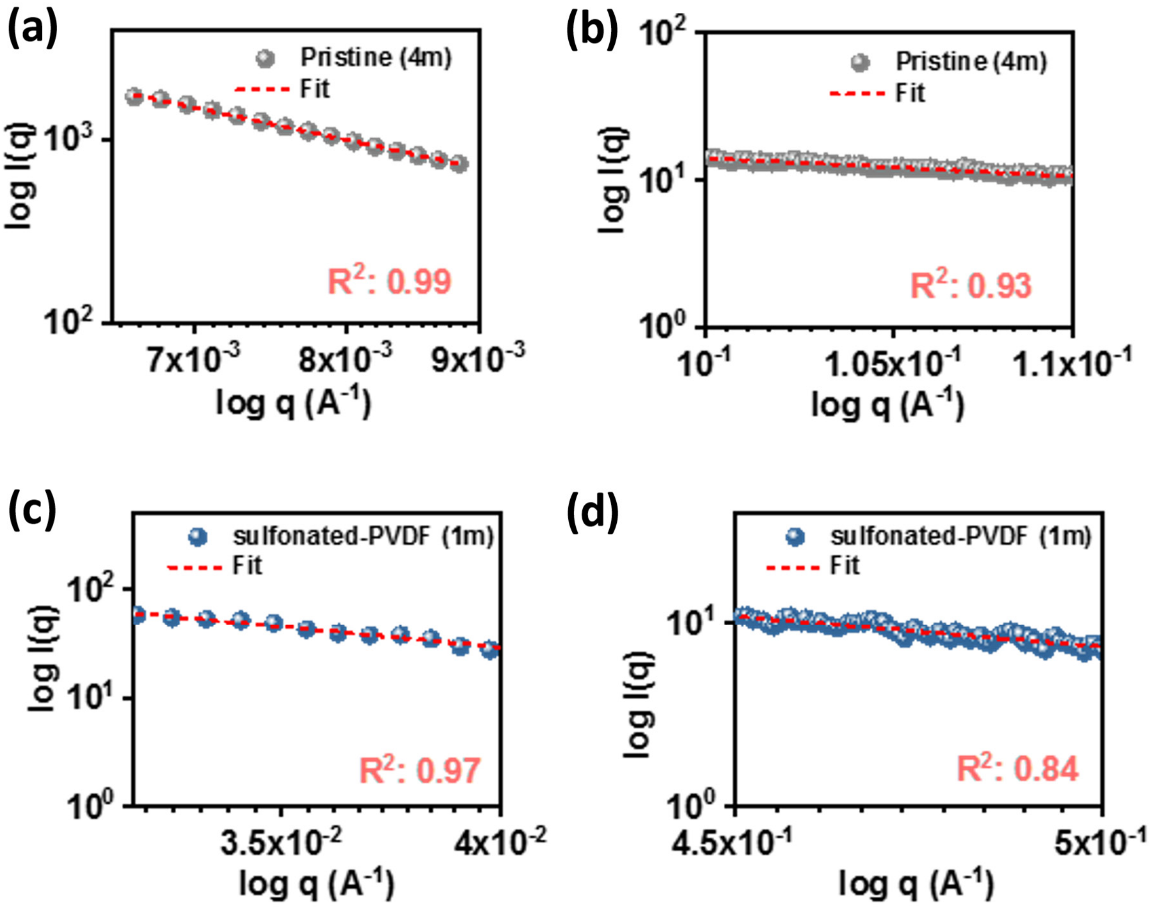

Figure S2. Log-log plot (a) region 1 of pristine PVDF-co-HFP membrane, (b) region 2 of pristine PVDF-co-HFP membrane, (c) region 1 of the sulfonated-PVDF-co-HFP membrane, and (d) region 2 of the sulfonated-PVDF-co-HFP membrane.

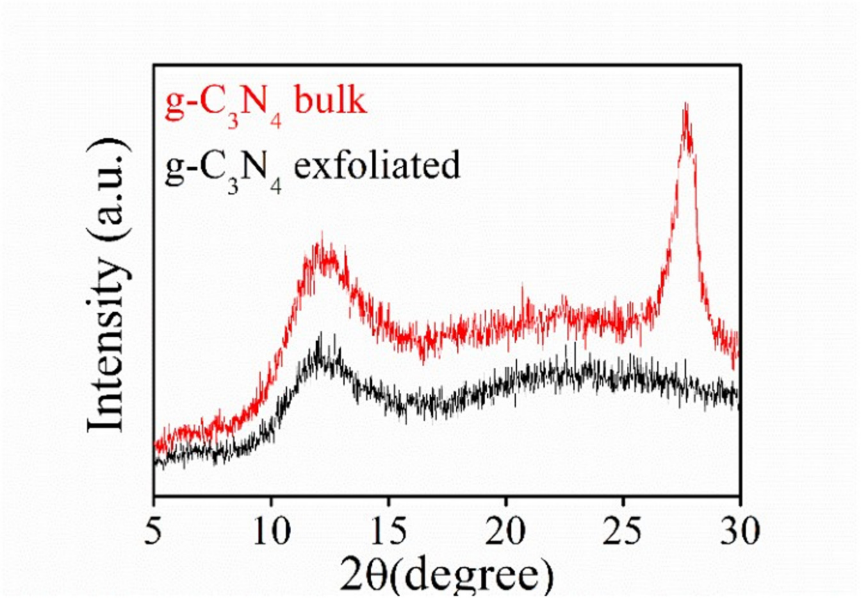

Figure S3 X-ray diffraction of the bulk nanoparticle and the exfoliated nanosheet of graphitic carbon nitride.

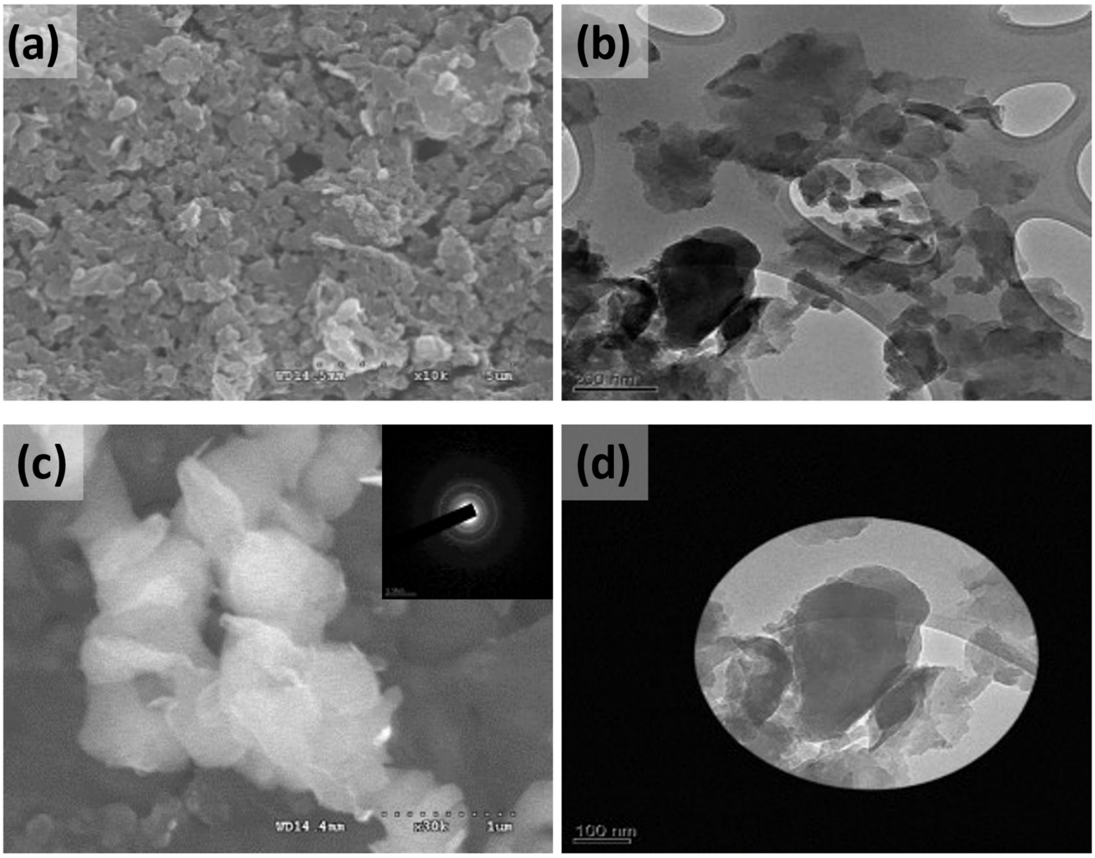

Figure S4 (a, b) SEM images of bulk and exfoliated g-C_3N_4, (c-d) TEM images of exfoliated g-C_3N_4.

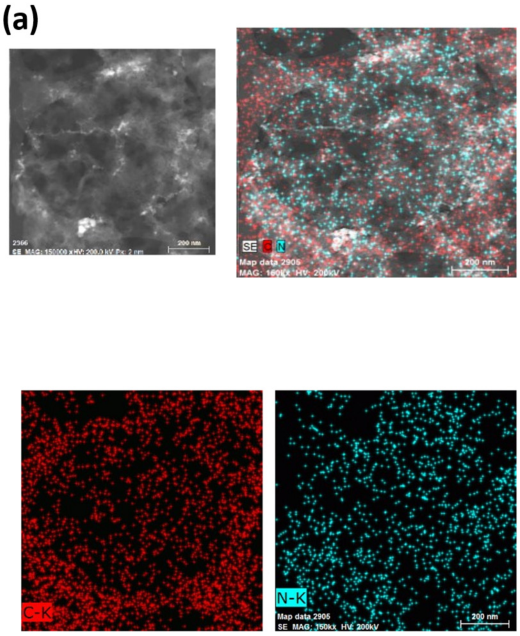

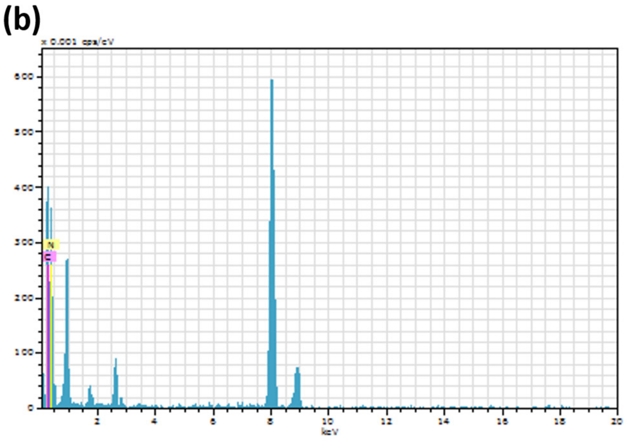

Element	Series	Carbon (wt %)	Atom (at%)
C	K	82.19	84.33
N	K	17.81	15.67

Figure S5 EDS analysis of a particular region of the particle, and (b) EDS analysis containing elemental composition for g-C_3N_4.

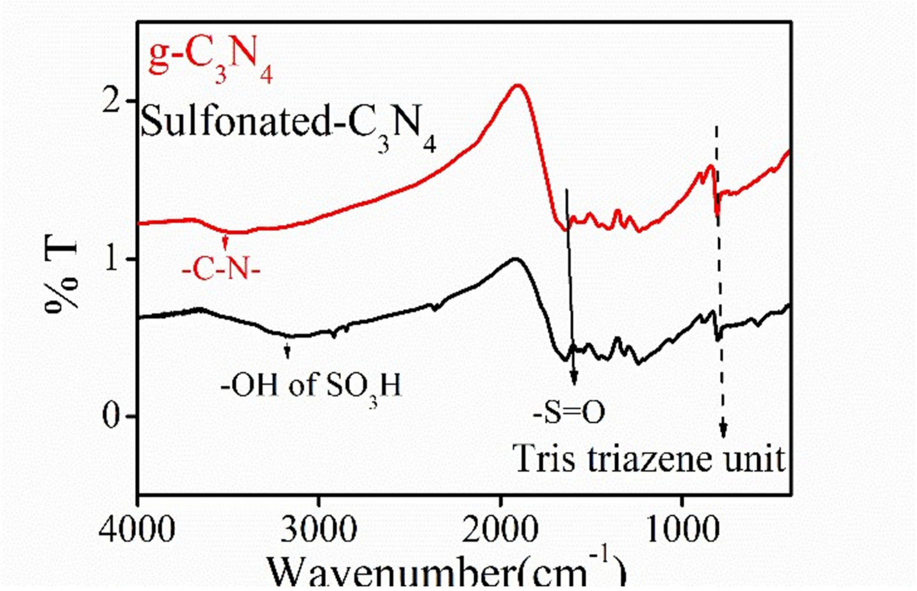

Figure S6 FT-IR spectra of pristine graphitic carbon nitride and sulfonated graphitic carbon nitride.

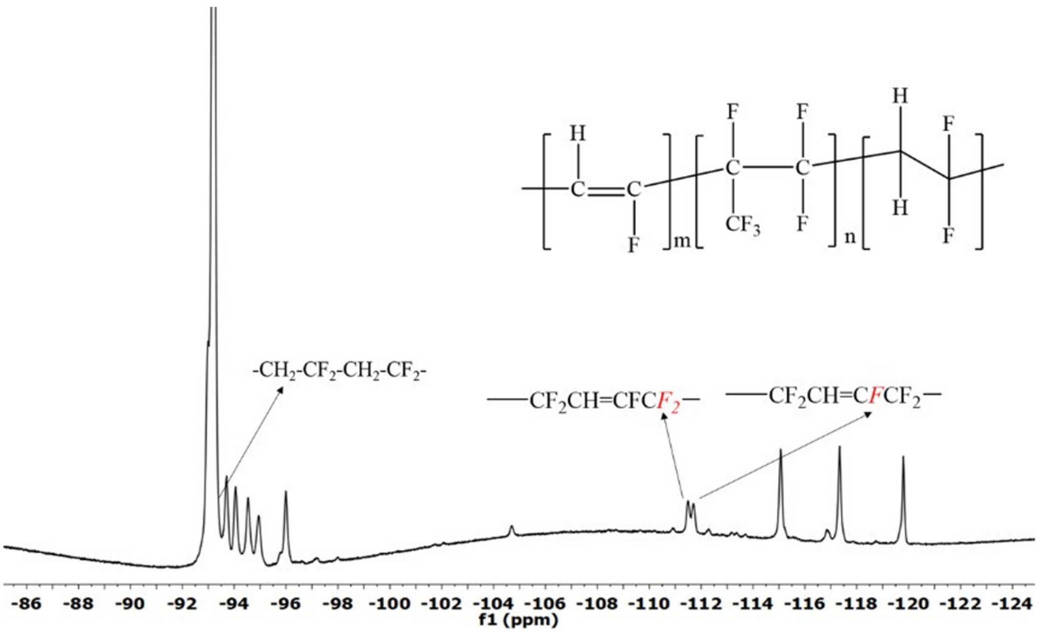

Figure S7 Fluorine-19 nuclear magnetic resonance spectroscopy (^{19}F- NMR) of dehydrofluorinated PVDF-HFP based ionomer.

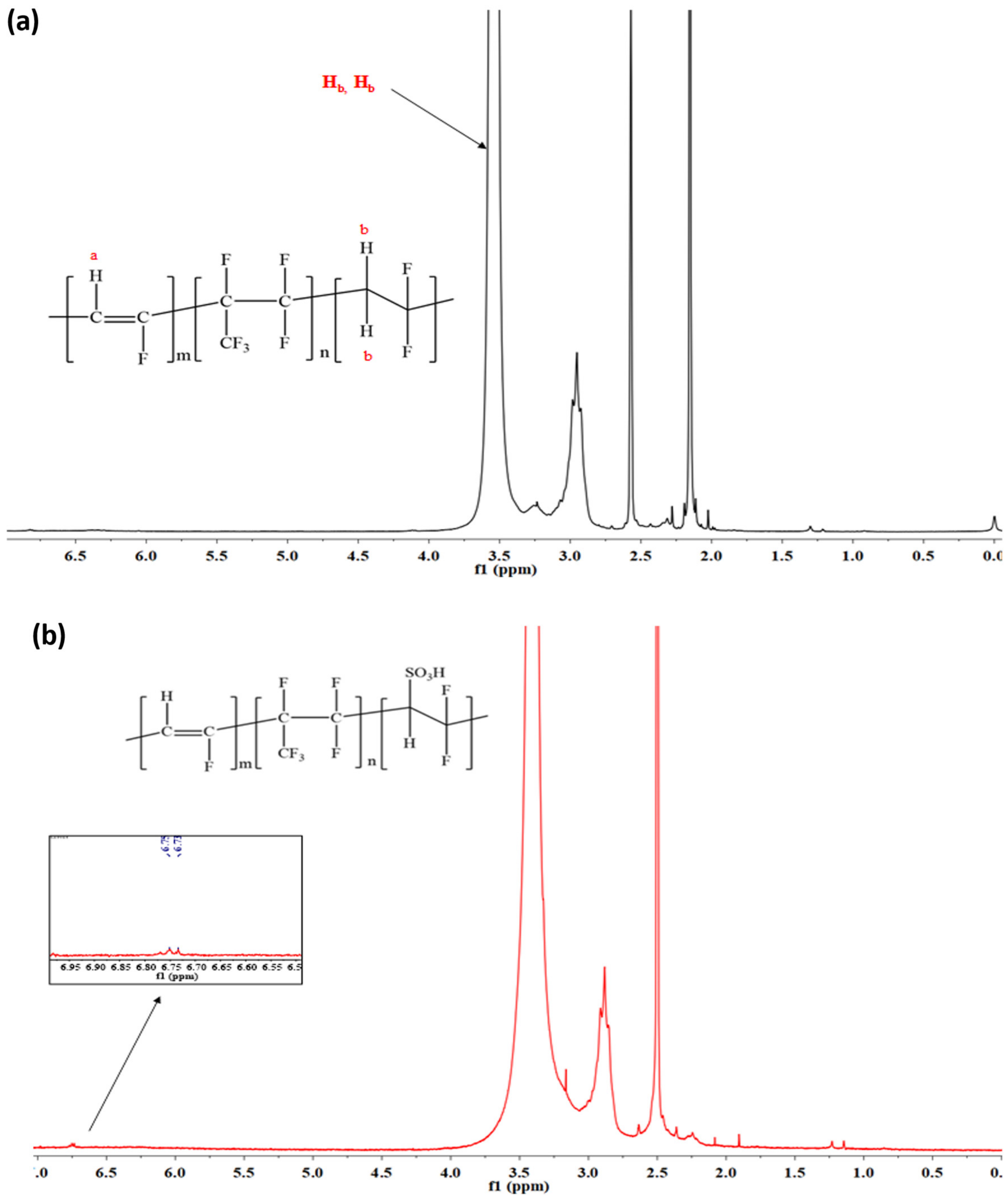

Figure S8 Proton Nuclear magnetic resonance spectroscopy (^{1}H-NMR): (a) dehydrofluorinated PVDF-HFP ionomer and (b) sulfonated-PVDF-HFP based ionomer.

Supplementary Note S2

3. Electrochemical and physicochemical characterization of the membrane

3.1. Water uptake

Water uptake is essential for the ion exchange membrane because it provides hydrophilicity of the membrane. There are two types of water present inside the membrane, which are mainly in the form of bound water and free water. Due to bound water, the transportation of ions takes place through the membrane. Graphitic carbon nitride has a polar group at its periphery, thus allowing the polymer to absorb water. Its hydrophilicity increases, which is a good sign for RED application because it reduces the concentration polarization interfaces during the experiment. The sulfonic acid group also tends to form hydrogen bonding with the water molecule, thus providing a hopping mechanism for the conduction of Na^+ ions.

The following equation calculates water uptake

$$WU(\%) = \frac{Wet_w - Dry_w}{Dry_w} X100 \ldots\ldots\ldots\ldots\ldots (1)$$

where, Wet_w is the weight of the wet membrane, and Dry_w is the weight of the dry membrane.

3.2. Ion exchange capacity and ionic conductivity

The ion exchange capacity of the cation exchange membrane is calculated by the acid-base titration method using phenolphthalein as an indicator. The first membrane was dipped into a 0.1 M NaCl solution for 12 hours so that the Na^+ ions exchange H^+. It was then titrated against 0.01 M NaOH solution to estimate ions get exchanged per gram weight of the membrane.

The equation that is used to calculate the ion exchange capacity is as follows:

$$IEC\ \left(\frac{meq}{g}\right) = \frac{V_{NaOH} X C_{NaOH}}{W_{dry}} \ldots\ldots\ldots\ldots\ldots\ldots (2)$$

Where, V_{NaOH} is the volume of NaOH used during the titration and C_{NaOH} is the concentration of standard sodium hydroxide solution, and W_{dry} is the weight of the dry membrane.

Ionic conductivity of prepared membranes was measured by AC impedance spectroscopy (Gamry potentiostat) at 25 ± 3^0C and calculated using equation 3. Before measuring, membranes were dipped into 1M H_2SO_4 aqueous solution for complete ionization of the sulfonic acid group.

$$\sigma = \frac{L}{RXA} \ldots\ldots\ldots\ldots (3)$$

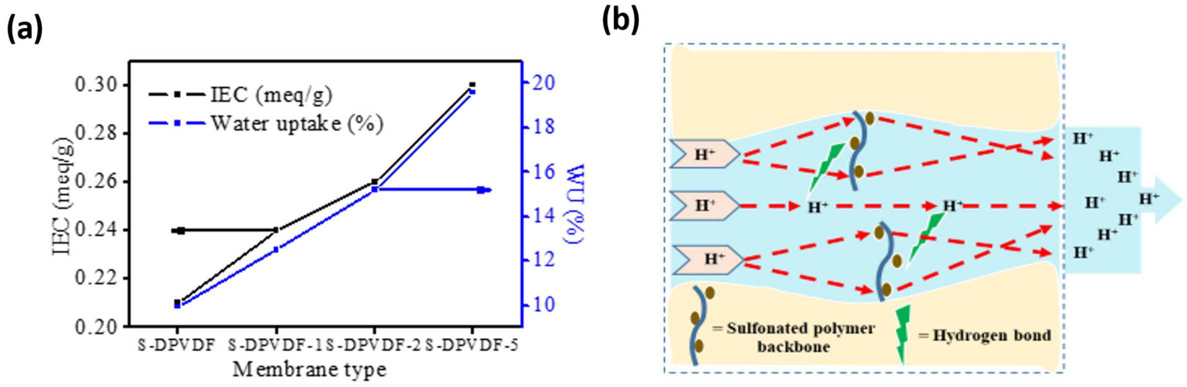

Figure S9 (a) Water uptake vs. Ion exchange capacity for different composite membrane, (b) Schematic illustration of ionic domains for the conduction of ions.

Table S3 Summary of physicochemical properties for commercial membranes and the s-PVDF developed in this work.

Specification	s-PVDF (This work)	Aquivion[2] Solvay	Nafion[3] N115 DuPont	Nafion[3] N117 DuPont	Hyflon[4] Solvay	Neosepta[5] CSE ASTOM	Selemion[6] CMV Asahi
Water uptake (%)	19.57	~35	38	38	45	25	22
Ion exchange capacity (meq g^{-1})	0.30	1.02	0.95-1.01	0.95-1.01	0.825	1.5-1.8	1.3-2.4
Conductivity (S cm^{-1})	$3.64x10^{-3}$ at 23 ^{o}C	0.02 at 50 ^{o}C, 50% RH	0.1 at 23 ^{o}C, 50% RH	0.1 at 23 ^{o}C, 50% RH	~0.1	1.8 (ohm cm^2) at 25°C NaCl sol	~0.006 in NH_4Cl sol
Thickness (μm)	~100	24.6 ± 0.7	127	183	63	160	137
Solvent	*N-methyl-2-pyrrolidone (NMP)*	*Isopropanol (IPA)*	*Isopropanol (IPA)*	*Isopropanol (IPA)*	-	-	-

Supplementary Note S3

4. RED stack Assembly

In our experiment, artificial sea and river water solutions were prepared, consisting of [0.6 M/0.01M] NaCl for concentrated and diluted compartments. The whole RED stack is composed of polymethylmethacrylate (PMMA) endplates with IEMs (CEM and AEM), silicon gaskets with spacers. The electrodes which were used during the RED experiment were made up of mixed metal oxides (MMO) containing Titanium (Ti) coated with Iridium (Ir) and Ruthenium (Ru). Both cations and anion exchange membrane were sandwiched between the endplates having an effective area of 49 cm^2. The rinse solution used in the RED experiment was prepared by mixing 0.05 M of $[Fe\ (CN)_6]^{-3}$/ $[Fe\ (CN)_6]^{-4}$ with 0.3 M NaCl. Both membranes were conditioned with 0.6 M NaCl solution for 12 h before use. A peristaltic pump (EMP-2000 W, EMS Tech) was used to recirculate solutions from three compartments: *viz* concentrated, diluted, and rinse in recirculation mode to RED stack. During the experiment, the spacers play a vital role in maintaining vertical material exchange and boundary formation.

The evaluation of RED performance was carried out by a 2410- Keithley Source Meter connected to electrodes. The electrochemical characteristics curve, i.e. (I-V) of RED stack in terms of power density, was obtained by the galvanostatic method obtained by measuring the terminal voltage while varying the current step 2mA per minute. The (I-V) characteristics were obtained to measure power density (Wm^{-2}) at three different flow rates for each membrane, i.e., 60 ml/min, 80 ml/min, and 100 ml/min. The maximum power density for the RED stack is measured by the following equation [7]:

$$P_{max} = \frac{U_{Stack} \cdot I_{Stack}}{A_{mem}} \quad \ldots\ldots (4)$$

Where P_{max} is the maximum power density of the stack (Wm^{-2}), U_{stack} is the voltage (V) generated by the membrane stack, I_{stack} is the scanned current (A), and A_{mem} is the effective area of the membrane.

4.1. Artificial solutions for RED experiment

Analytical grade sodium chloride (NaCl) was used to prepare a model solution of seawater with a concentration (0.6 M of NaCl) while a concentration of (0.01 M of NaCl) was used to prepare a model solution of river water. As discussed above, these solutions were passed in a single recirculation mode through the RED stack by a peristaltic pump, and a linear flow rate was varied from 60 ml min^{-1} to 100 ml min^{-1}. All experiments were conducted at $25^0C \pm 3^0C$.

Table S4. Summary of the power density for commercially available membranes and the sPVDF/Neosepta membrane used in this work. Here, *A*: Active membrane area; N: Number of cell pairs; $P_{d,max}$: Maximum power density.

Type	Area (cm²)	N	Pmax (W/m²)	Ref
sPVDF/Neosepta	7x7	1	0.2	This work
Neosepta CMX (Tokuyama)/Fuji T1 AEM (Fujifilm)	10x10	5	~0.2*	[8]
Ralex CMH/AMH, (MEGA)	10x10	5	~0.26*	[9]
Neosepta AMX/CMX (Tokuyama)	10x10	10	~0.42*	[9]
Fuji T1 CEM/Fuji T1 AEM (Fujifilm)	6.5x6.5	10	~0.7*	[10]
Neosepta ACS/CMS (Tokuyama)	10x10	5	3.8	[11]
FumasTech FAD/FKD (GmbH)	10x10	50	6.7 (60 °C)	[11]
Qianqiu AEM/CEM, (Hangzhou Qianqiu)	25x75	25	~0.83	[12]
Selemion AMV/CMV (Asahi Glass)	10x10	5	1.18	[13]
FumaTep FKD and FAD (GmbH)	10x10	25	1.17	[13]
FumaTep FKD and FAD (GmbH)	10x10	50	0.93	[14]
Qianqiu Heterogeneous AEM/CEM (Hangzhou QianQiu)	10x10	5	1.05	[13]
Fuji AEM/CEM (Fujifilm)	10x10	25	1.06	[15]
PC-SK and PC-SA (PCCell, Germany)	10x10	10	~0.33	[7]
Neosepta AMX/CMX (Tokuyama)	100	3	0.87	[16]
Neosepta CMX/AMX (Tokuyama	6x13	5	0.59	[17]
FKS/FAS (FumaTech GmbH)	10x10	5	2.2	[18]
FKS/FAS (FumaTech GmbH)	10x10	5	0.5	[18]
Neosepta CMX/AMX (Tokuyama)	10x10	2-30	0.95	[19]
Ralex CMH- PES/AMH-PES (MEGA)	5x5	5	0.62	[20]
Ralex CMH- PES/AMH-PES (MEGA)	10x10	5	0.65	[20]

Supplementary References

1. Rubatat, L.; Rollet, A. L.; Gebel, G.; Diat, O., Evidence of elongated polymeric aggregates in Nafion. *Macromolecules* **2002,** *35* (10), 4050-4055.
2. Woo, S. H.; Taguet, A.; Otazaghine, B.; Mosdale, A.; Rigacci, A.; Beauger, C., Physicochemical properties of Aquivion/fluorine grafted sepiolite electrolyte membranes for use in PEMFC. *Electrochimica Acta* **2019,** *319*, 933-946.
3. Membranes, D. N. P., http://thenanoholdings.com/data/goodsImages/GOODS1_1400494306.pdf.
4. Ghielmi, A.; Vaccarono, P.; Troglia, C.; Arcella, V., Proton exchange membranes based on the short-side-chain perfluorinated ionomer. *Journal of Power Sources* **2005,** *145* (2), 108-115.
5. corp, A., http://www.astom-corp.jp/en/product/10.html.
6. Choi, J.-H.; Kim, S.-H.; Moon, S.-H., Heterogeneity of Ion-Exchange Membranes: The Effects of Membrane Heterogeneity on Transport Properties. *Journal of Colloid and Interface Science* **2001,** *241* (1), 120-126.
7. Zhu, X. P.; He, W. H.; Logan, B. E., Reducing pumping energy by using different flow rates of high and low concentration solutions in reverse electrodialysis cells. *J. Membr. Sci.* **2015,** *486*, 215-221.
8. Moreno, J.; Diez, V.; Saakes, M.; Nijmeijer, K., Mitigation of the effects of multivalent ion transport in reverse electrodialysis. *J. Membr. Sci.* **2018,** *550*, 155-162.
9. Vermaas, D. A.; Veerman, J.; Saakes, M.; Nijmeijer, K., Influence of multivalent ions on renewable energy generation in reverse electrodialysis. *Energy Environ. Sci.* **2014,** *7* (4), 1434-1445.
10. Rijnaarts, T.; Huerta, E.; van Baak, W.; Nijmeijer, K., Effect of Divalent Cations on RED Performance and Cation Exchange Membrane Selection to Enhance Power Densities. *Environ Sci Technol* **2017,** *51* (21), 13028-13035.
11. Daniilidis, A.; Vermaas, D. A.; Herber, R.; Nijmeijer, K., Experimentally obtainable energy from mixing river water, seawater or brines with reverse electrodialysis. *Renew Energ* **2014,** *64*, 123-131.
12. Veerman, J.; Saakes, M.; Metz, S. J.; Harmsen, G. J., Electrical power from sea and river water by reverse electrodialysis: a first step from the laboratory to a real power plant. *Environ Sci Technol* **2010,** *44* (23), 9207-12.
13. Veerman, J.; de Jong, R. M.; Saakes, M.; Metz, S. J.; Harmsen, G. J., Reverse electrodialysis: Comparison of six commercial membrane pairs on the thermodynamic efficiency and power density. *J. Membr. Sci.* **2009,** *343* (1-2), 7-15.
14. Veerman, J.; Saakes, M.; Metz, S. J.; Harmsen, G. J., Reverse electrodialysis: Performance of a stack with 50 cells on the mixing of sea and river water. *J. Membr. Sci.* **2009,** *327* (1-2), 136-144.
15. Avci, A. H.; Sarkar, P.; Tufa, R. A.; Messana, D.; Argurio, P.; Fontananova, E.; Di Profio, G.; Curcio, E., Effect of Mg2+ ions on energy generation by Reverse Electrodialysis. *J. Membr. Sci.* **2016,** *520*, 499-506.
16. Dlugolecki, P.; Gambier, A.; Nijmeijer, K.; Wessling, M., Practical potential of reverse electrodialysis as process for sustainable energy generation. *Environ Sci Technol* **2009,** *43* (17), 6888-94.
17. Zhang, B.; Gao, H.; Chen, Y., Enhanced Ionic Conductivity and Power Generation Using Ion-Exchange Resin Beads in a Reverse-Electrodialysis Stack. *Environ Sci Technol* **2015,** *49* (24), 14717-24.
18. Vermaas, D. A.; Saakes, M.; Nijmeijer, K., Doubled Power Density from Salinity Gradients at Reduced Intermembrane Distance. *Environmental Science & Technology* **2011,** *45* (16), 7089-7095.
19. Vermaas, D. A.; Bajracharya, S.; Sales, B. B.; Saakes, M.; Hamelers, B.; Nijmeijer, K., Clean energy generation using capacitive electrodes in reverse electrodialysis. *Energy Environ. Sci.* **2013,** *6* (2), 643-651.
20. Vermaas, D. A.; Saakes, M.; Nijmeijer, K., Enhanced mixing in the diffusive boundary layer for energy generation in reverse electrodialysis. *J. Membr. Sci.* **2014,** *453*, 312-319.
